# Forty years of a Postgraduate Program in Cardiology at a Brazilian public university: indicators of its graduates

**DOI:** 10.1590/acb394824

**Published:** 2024-08-05

**Authors:** Carlos Eduardo Braga, Talita Dias da Silva, Claudia Joanete da Silva, Cleidivan Alves dos Santos, Frank Menezes Rodrigues, Adriano Henrique Pereira Barbosa, Adriano Caixeta, Maria Cristina de Oliveira Izar, Bráulio Luna-Filho, Francisco Antonio Helfenstein Fonseca, Fernando Sabia Tallo, Leonardo Roever, Afonso Caricati-Neto, Francisco Sandro Menezes-Rodrigues

**Affiliations:** 1 Universidade Federal de São Paulo – São Paulo (SP) – Brazil.; 2 Universidade Federal do Delta do Parnaíba – Parnaíba (PI) – Brazil.; 3 Universidade Metodista de São Paulo – São Bernardo do Campo (SP) – Brazil.; 4 Universidade Federal de Uberlândia – Uberlândia (MG) – Brazil.

**Keywords:** Health Postgraduate Programs, Cardiology, Students, Education, Graduate

## Abstract

**Purpose:**

To evaluate the profile of graduates of the Postgraduate Program (PGP) in Cardiology of a public federal university, according to sociodemographic factors and professional trajectory.

**Methods:**

The variables were collected from databases from the observed institution and digital platforms. The analysis of differences between the various levels of degrees was carried out in three cohorts: the entire historical series (graduates from 1978–2021), the first 20 years (1978–1997) and the second 20 years (1998–2018).

**Results:**

The results demonstrated that most students from the PGP completed a PhD and are men over 30 years old, they came from public universities and the Southeast region. In the first 20 years, significant differences were observed in the distribution of masters and doctors working professionally at the institution analyzed, as well as in the age of the students. In the 20 years of the second half, there were differences between masters and PhD working professionally in the institution itself, as they came from private universities, they are women and PhD.

**Conclusions:**

The changes in the profile of masters and PhD that graduated from this PGP in cardiology reflect transformations that occurred in the job market and academy over the decades.

## Introduction

The last few years have been characterized by the high competitiveness in all sectors of the job market, which shows even more the need for qualified professionals. In addition, the growing number of recent graduates has led to the extension of academic life and, consequently, the amplification of the number of post-graduate courses^1^. *Stricto sensu*, the post-graduate programs (PGP), whether at the masters or PhD degree, have objectives connected to the scientific formation of researchers and teachers^2^. Despite that, it isn’t uncommon that PGPs of this type are attractive for professionals with different ambitions and motivations^1^^,^ 3.

The PGP in Cardiology from the Brazilian public university, the object of this study, was introduced in 1975. In the beginning, only medical students were accepted, however, since 1999, the program was reformulated to allow the entry of non-medical students, contributing to its interdisciplinarity. Throughout its more than 40 years of existence, the program graduated hundreds of masters, PhD, and post-PhD, that act in numerous institutions in and outside of Brazil^4^.

Indicators obtained in 2010 showed that the PGP in Cardiology is a program with a high rate of publications, good academic performance, and satisfaction on the part of graduates. In a study published by Brock et al.^4^, sociodemographic and professional characteristics of the graduates up to that time were evaluated, a profile of masters and PhDs that in their majority were men with over 30 years, with medical education and from the Southeast.

Continuously evaluating the profile and professional trajectory of the graduates can help PGP adapt their curricula. The objective of this study was to analyze the profile of graduates in a PGP in Cardiology of a public federal university since its foundation, evaluating the differences between degree levels throughout the program’s history.

## Methods

### Data collection

The records of graduates of the PGP in Cardiology were obtained through the institution’s database, like the Integrated University Information System, Office of Institutional Strategic Data (e Dados), as well as records from the PGP itself. This information was confronted with data available in digital platforms: Lattes, LinkedIn, National Register of Health Establishments, and National Register of Higher Education Courses and Institutions (Cadastro e-MEC). This study was conducted in accordance with Resolution no. 466/2012, and approved in 2020 by the Research Ethics Committee of the Universidade Federal de São Paulo (under the protocol no. #6688300120).

### Collected variables

Quantitative variables “Age of entry” and “Age of exit” were extracted, calculated as the age (in years) of the student at entry and exit from the post-graduate course. “Duration of the course” represents the time in months from enrolment to completion of the course. The qualitative variables extracted were: “Professional performance in the institution”, according to the institution in which the graduate was inserted in the moment of the data collection; “Type of undergraduate institution” (public or private university); “Gender” (male or female); “Region of origin”, according to the graduates’ state of birth; and “Undergraduate course”, referring to the bachelor’s degree.

### Data analysis

Student data was analyzed according to the level of the completed course (masters, PhD or post-doctorate). In face of the important transformations that occurred during the history of the PGP in Cardiology, the analyses were divided in cohorts according to the year of entry of the students:

- The entire historical series, covering graduates between 1978 and 2021;
- The first 20 years, covering graduates from 1978 to 1997;
- The second 20 years, graduates from 1998 to 2018.

By having a reduced number of representatives (n = 11), the data of post-doc graduates were only analyzed in the cohort considering the entire historical series.

The normality of the variables was tested using the Kolmogorov-Smirnov’s test, making possible the utilization of parametric tests. Analysis of variance (ANOVA) was used to compare quantitative factors, while the χ^2^ test was useful in the analysis of qualitative factors. The test of equality of two proportions was applied to characterize the distribution of the qualitative factors and to compare whether the proportion of responses to the variables and their levels was statistically significant. The level of significance was established in 0.05.

## Results

### Historical series (1978–2021)

In the historical series (1978–2021), 210 masters’ degrees, 214 PhDs and 11 post-doctor were completed in the PGP in Cardiology. The masters’ degree’s students had a lower average age in comparison to the post-doc and PhDs. The PhD level had the biggest course duration in comparison to the masters’ degree.

By covering all levels, it was observed that most of the sample was composed by graduates without acting professionally in their own institution (71.5%), males (58.9%), born in the Southeast region (67.6%), medical graduates (70.1%) and by public universities (63%). There were differences in gender distribution between the levels, with a higher proportion of women amongst the masters’ degree graduates. There were differences in the distribution of professionals acting in the studied organization and from private universities, with a higher proportion of these being represented in the post-doc. The distribution of graduation courses was also different between the levels, with greater presence of PhDs amongst PhDs (Table 1).

**Table 1:** Sociodemographic data and professional trajectory of graduates from a Post-graduate Program in Cardiology at a Brazilian public university.

Sociodemographic data	Entire historical series 1978–2021					First 20 years 1978–1997				Second 20 years 1998–2018		
	MScs	PhD	Post-Doc	*p*-value		MScs	PhD	*p*-value		MScs	PhD	*p*-value
**Entry age** (years, average)	33.4	34.9	39.6*	< 0.01		30.4	34	< 0.01		34.5	35.1	0.54
**Exit age** (years, average)	36.9† ‡	39.1*	42.8*	< 0.01		34.3	37.9	< 0.01		37.7	39.3	0.11
**Duration of the course** (months, average)	41.8†	51.0* ‡	38.4	< 0.01		47.3	46.7	0.92		37.7	49.8	< 0.01
**Working in their own institution % (n)**												
Yes	22.9 (48)	32.7 (70)	54.5 (6)	0.013		28.8 (17)	47.7 (21)	0.049		16.7 (21)	28.6 (44)	0.019
No	76.7 (161)	67.3 (144)	45.5 (5)			71.2 (42)	52.3 (23)			83.3 (105)	71.4 (110)	
**Undergraduate institution % (n)**												
Public	59 (124)	68.2 (146)	36.4 (4)	0.032		78.3 (47)	81.8 (36)	0.66		54.8 (68)	67.5 (104)	0.03
Private	40 (84)	31.8 (68)	63.6 (7)			21.7 (13)	18.2 (8)			45.2 (56)	32.5 (50)	
**Gender %** (n)												
Female	46.7 (98)	36.9 (79)	18.2 (2)	0.037		21.7 (13)	13.6 (6)	0.29		58.7 (74)	43.5 (67)	0.011
Male	53.3 (112)	63.1 (135)	81.8 (9)			78.3 (47)	86.4 (38)			41.3 (52)	56.5 (87)	
**Region of origin % (n)**												
Southeast	66.2 (139)	68.2 (146)	81.8 (9)	0.42		73.3 (44)	72.7 (32)	0.91		59.5 (75)	66.2 (102)	0.23
Northeast	12.4 (26)	14 (30)	18.2 (2)			6.7 (4)	9.1 (4)			16.7 (21)	16.2 (25)	
North	13.3 (28)	7.5 (16)	-			8.3 (5)	4,5 (2)			17,5 (22)	8,4 (13)	
Central-West	2.4 (5)	4.2 (9)	-			5 (3)	4.5 (2)			1.6 (2)	4.5 (7)	
South	3.3 (7)	5.1 (11)	-			3.3 (2)	6.8 (3)			4 (5)	3.9 (6)	
Abroad	2.4 (5)	0.9 (2)	-			3.3 (2)	2.3 (1)			0.8 (1)	0.6 (1)	
**Undergraduate course % (n)**												
Medicine	66.2 (139)	75.2 (161)	45.5 (5)	< 0.01		100 (60)	100 (44)	-		55.6 (70)	71.4 (110)	< 0.01
Physiotherapy	6.7 (14)	12.6 (27)	9.1 (1)			-	-			8.7 (11)	14.9 (23)	
Physical education	6.2 (13)	4.2 (9)	36.4 (4)			-	-			7.9 (10)	4.5 (7)	
Nursing	5.7 (12)	2.8 (6)	-			-	-			7.1 (9)	3.2 (5)	
Nutrition	5.2 (11)	0.9 (2)	-			-	-			7,9 (10)	0,6 (1)	
Biomedicine	4.3 (9)	0.5 (1)	-			-	-			5,6 (7)	0,6 (1)	
Veterinary medicine	1.4 (3)	1.4 (3)	-			-	-			2.4 (3)	1.2 (2)	
Psychology	1.4% (3)	0.5٪ (1)	-			-	-			2.4٪ (3)	0.6٪ (1)	
Radiology technology	1.9 (4)	-	-			-	-			1,6 (2)	-	
Others@	1 (2)	2 (4)	0.1 (1)			-	-			0.6 (1)	2.4 (4)	

* *p* < 0.05 vs. masters;

† *p* < 0.05 vs. PhD

‡ *p* < 0.05 vs. post-doctor;

values in bold denote statistical significance;

@ biological sciences, pharmacy, data processing, occupational therapy, and mathematics.

Source: Elaborated by the authors.

### First 20 years (1978–1997)

Sixty masters and 40 PhD were concluded during the first 20 years of existence of the explored PGP in Cardiology. The mean entry and exit age were significantly higher amongst PhDs in comparison to masters. There were no significant differences between degree levels for the duration of the course. Many graduates during this period didn’t act professionally in the university itself (36.9%), but there was a significantly greater proportion of them between the PhD students. Most of the graduates were males (81.7%), from public universities (79.8%) and born in the Southeast Region (73.1%), without significant differences between degree levels. Medical graduates were the only represented in this sample (Table 1).

### Second 20 years (1998–2018)

In the second 20 years (1998–2018), 126 masters and 154 PhDs were concluded during this period. There were no significant differences between the degree levels for age of entry and exit. The duration of the PhD course was significantly greater than that of the maters’ degree. Similarly, to what was observed in the first 20 years, a minority of graduates didn’t act professionally in the institution itself (23.2%). There was also a greater proportion of PhD graduates acting in the institution in comparison to masters. Most of graduates had a medical degree (64.3%) and came from public universities (54.8%).

When separating between degree levels, there were differences in the distribution of graduates from public universities and medical graduates, most of whom are at the PhD level. Despite the proportion of men and women being practically equal when considering all levels, there was a significantly higher quantity of women at the master level than at the PhD level. Many graduates were from the Southeast (63.2%), without significant differences in relationship to the distribution between the different degree levels (Table 1).

## Discussion

The PGP passed through important changes in the last decades, passing several national policies related to education and research, as well as the evolution of the academic world and the job market. When evaluating the graduate’s profile in the PGP in Cardiology of the studied Brazilian federal university throughout 40 years of existence, changes were observed in the sociodemographic characteristics, such as age and gender, in addition to professional trajectory, like course duration, academic background in private universities, and professional acting in the university.

Age at entitlement can be an important maker to evaluate for how long highly qualified individuals can exert productive activities. The National Plan of Post-Graduation of 2011 to 2020 classifies post-graduation as “long and late”, and recommends a reversal to “lower age levels, so that there is sufficient renewal and longevity to meet the country’s needs”^5^. However, it didn’t occur in the PGP in Cardiology of this federal public university. From the second 20 years onward, the mean age of masters’ graduates has risen by more than three years and of doctors by almost two years. By observing the indicators of evaluated graduates in the past decade, it is possible to notice that this trend has been intensifying ever since — in 2010, the mean masters graduate age was 35.4 years old, and doctors of 38.2^4^. The relatively high age of entry and exit can be due to the majority of graduates being physicians, professionals who tend to be older when looking for PGP^1^^,^ 6.

If in the first 20 years of the PGP most students were composed of men, in the second 20 years the proportion became practically equal. This change agrees with data of Latin America and of the world, which show that women have become the majority of post-graduate students in the last decades^7^. However, there is a significant difference in the distribution between degrees. While women are majority amongst masters, in the doctorate degree they represent a little less than half of graduates. One of the possible explanations for this result may be the majority of graduates is supposedly composed by cardiologists, a population in which it is estimated that there are fewer than 30% women^8^.

Many students of the PGP were from the Southeast, regardless the degree level and course completion period. Numerically, there was an increase in the proportion of students coming from the North and Northeast in the second 20 years in comparison to the previous decades. Together with this result, another study with these graduates of this PGP showed that approximately half of the students returned to their institution of origin, which makes the program a potential exporter of highly qualified professionals to various regions of the country^4^.

The average time to obtain a title can have a great impact in the evaluation of PGPs^9^. In this PGP, this metric only became statistically different between the courses from the second 20 years onwards: the mean time of conclusion of masters was of 37.7 months, while for doctors it was 49.8 months. The Coordination for the Improvement of Higher Education Personnel (CAPES) sets 24 months as the standard for master’s degree courses and 48 months for doctorate courses^10^. The efficiency of master’s degree is less than ideal, while that of doctors is close to what is expected. The efficiency of formation can significantly impact the distribution of CAPES’ scholarships, which affects the scientific production and the PGP’s metrics^11^. The motivations and satisfactions with the course can be an important measure for the PGPs to adapt their academic curricula and selection, to ensure the program’s attractiveness for potential new students and trace new strategies to improve its evaluation metrics^12^.

The examined PGP in cardiology only offers *stricto sensu* courses, traditionally focused in the academic formation and with focus on scientific production^2^^,^ 4 ^,^ 13. Despite this type of PGP having the formation of teachers and researchers as a principle, the motivation for the choice of these programs goes beyond academic vocation^1^. In this study, almost half of graduates until 1997 was active professionally in their own institution, while in the second 20 years this proportion decreased to one third of graduates. This difference may reflect the profile of the incoming students and the university culture at the time: in 1970, part of the reason for the emergence of PGPs *stricto sensu* in the medical area involves the interest in improving the institution’s own clinical staff, and, this way, good part of the students already had acted professionally at the university prior to the creation of the program. In the same way as this medical school, other leading cardiology institutions saw the necessity of creating PGPs as a way of preparing its medical staff to exert high-level didactic and scientific activities^12^^,^ 14. With the establishment and expansion of the program, it is expected that students with different professional motivations are interested by the education offered by the PGP, thus diversifying the profile of graduates and their professional trajectory^12^.

The difference in the professional profile of the graduates becomes evident when evaluating their undergraduate courses. In the second 20 years, despite most students still being made up by doctors, professionals such as physiotherapists, physical educators and nurses made up a third of the program’s graduates. This result isn’t surprising when considering that cardiology is a specialty of great interdisciplinarity according to CAPES^13^. This PGP, like other programs turned to medical specialties, has a much more clinical focus, possibly explaining the greater attraction of professionals with degrees related to care^14^^,^ 15.

Most of the graduates were from public universities. However, it was observed a numerical increase in the proportion of students coming from private institutions in the second 20 years, especially between the master’s graduates. This change reflects the expansion of higher education that has taken place in recent decades, especially in private institutions, who currently educates the majority of recent graduates^16^. Despite this data, public institutions continue being leaders in research, citation impacts and relationships with industry^17^. With this, it is possible that public post-graduate courses, like the PGP in this study, be attractive to students coming from private universities seeking a more robust scientific formation.

In summary, the results show that the profile of graduates of the PGP in Cardiology differed according to the level of degree and the period in which the course was completed. During the more than 40 years of history of this PGP in Cardiology, important changes occurred related to the greater participation of women in academy, greater proportion of students coming from private universities and non-medical students, as well as the diversification of the professional destination of graduates.

## Conclusion

The PGP in Cardiology of this public Brazilian university suffered important changes over 40 years of history. Over the decades, the sociodemographic and professional profile of the students accompanied some of the changes that have occurred at university level, of the country and the world, diversifying the characteristics of masters and PhDs trained by this PGP.

## Data Availability

All data sets were generated or analyzed in the current study.
